# Proteolysis targeting chimeras (PROTACs) in cancer therapy

**DOI:** 10.1186/s13046-020-01672-1

**Published:** 2020-09-15

**Authors:** Alberto Ocaña, Atanasio Pandiella

**Affiliations:** 1 Experimental Therapeutics Unit, Medical Oncology Department, Hospital Clínico San Carlos, and IdISSC, Madrid, Spain; 2 Centro de Investigación Biomédica en Red Oncología (CIBERONC), Madrid, Spain; 3 Centro Regional de Investigaciones Biomédicas, Castilla-La Mancha University (UCLM), Albacete, Spain; 4 IBMCC-CSIC and IBSAL, Salamanca, Spain

**Keywords:** PROTACs, BET inhibitors, Proteasome, Ubiquitination, Protein degradation

## Abstract

Exploitation of the protein degradation machinery as a therapeutic strategy to degrade oncogenic proteins is experiencing revolutionary advances with the development of proteolysis targeting chimeras (PROTACs). PROTACs are heterobifunctional structures consisting of a ligand that binds a protein to be degraded and a ligand for an E3 ubiquitin ligase. The bridging between the protein of interest and the E3 ligase mediated by the PROTAC facilitates ubiquitination of the protein and its proteasomal degradation. In this review we discuss the molecular medicine behind PROTAC mechanism of action, with special emphasis on recent developments and their potential translation to the clinical setting.

## Novel druggable vulnerabilities in cancer

Cancer is a multistep process in which genomic and epigenomic alterations lead to the abnormal cellular proliferation and dissemination [1]. Identification of molecular vulnerabilities that maintain the oncogenic phenotype has attracted major interest as the first step for the development of novel therapeutics [2].

A disbalance in the homeostasis of the protein production can be an oncogenic vulnerability in some tumors [3, 4], as demonstrated by the arrival of proteasome inhibitors to the oncology clinic [3, 4]. A novel class of agents that exploit the cellular protein degradation machinery with therapeutic purposes are the Proteolysis Targeting Chimeras or PROTACs [5]. These compounds can be used to facilitate proteasomal degradation of proteins that participate in the prooncogenic process. Importantly, PROTACs can be used to target a variety of proteins, including those with enzymatic activity or others difficult to target, such as those with scaffolding properties [3]. That is the case of transcription factors (TFs) (see glossary), which represent a large family of proteins against which very limited therapeutic options exist [6, 7]. TFs, as well as nuclear receptors, have been involved in the oncogenic generation of several malignancies. In fact, genomic alterations in *c-MYC, FOXO1* or the androgen receptor *(AR)* have been described in neuroblastoma, breast or prostate cancer, respectively [6, 8]. A therapeutic strategy that has been contemplated is the reduction of the expression of these proteins by inducing their degradation. In this context, two PROTACs targeting the AR and estrogen receptor (ER) have reached the clinical setting being explored in two phase I studies in prostate and estrogen receptor-positive breast cancer [9].

In this review we will focus on strategies to increase protein degradation of druggable and undruggable targets focusing on PROTACs, describing the current stage, the main limitations and their potential for improvement.

## Targeting protein degradation

Cell homeostasis depends on an accurate control of the quantity and quality of constituent proteins [10, 11]. This is more necessary in cells that have a high rate of turnover as they need to synthesize and consequently degrade proteins in an efficient manner [10, 11]. Protein degradation may occur in the lysosomes, by the action of acidic proteases that degrade proteins reaching these organelles [12]. Such situation is observed in the case of membrane receptors, that may increase their internalization and targeting to the lysosomes upon activation by their ligands or after binding to anti-receptor antibodies. This characteristic is exploited in the case of therapeutic antibody-drug conjugates (ADCs), a sophisticated evolution of anti-receptor antibodies. ADCs are composed of three components: an antibody against a protein expressed on the surface of tumor cells, a chemotherapeutic agent, and a linker that binds both. Upon interaction with the cell surface protein, the latter is driven to the endocytic lysosomal route. In the lysosomes, the ADC is proteolytically degraded facilitating the release of membrane-permeant cytotoxic drugs outside the lysosome to reach its cellular target. Acting on the lysosome has been less explored, although some recent preclinical studies have demonstrated the potential utility of this strategy, at least for the treatment of some proteinopathies [13].

Another major proteolytic system is the ubiquitin-proteasome degradation pathway, which relies on the ubiquitination of proteins, a process that triggers their degradation [14]. The targeting of an oncogenic protein or a protein not essential for the oncogenic phenotype but critical in the proliferation process, may also result in clinical benefit. This strategy needs ubiquitination of a target protein to subsequently be recognized by the proteasome [15].

## Protein degradation and the ubiquitination system

**Fig. 1:**
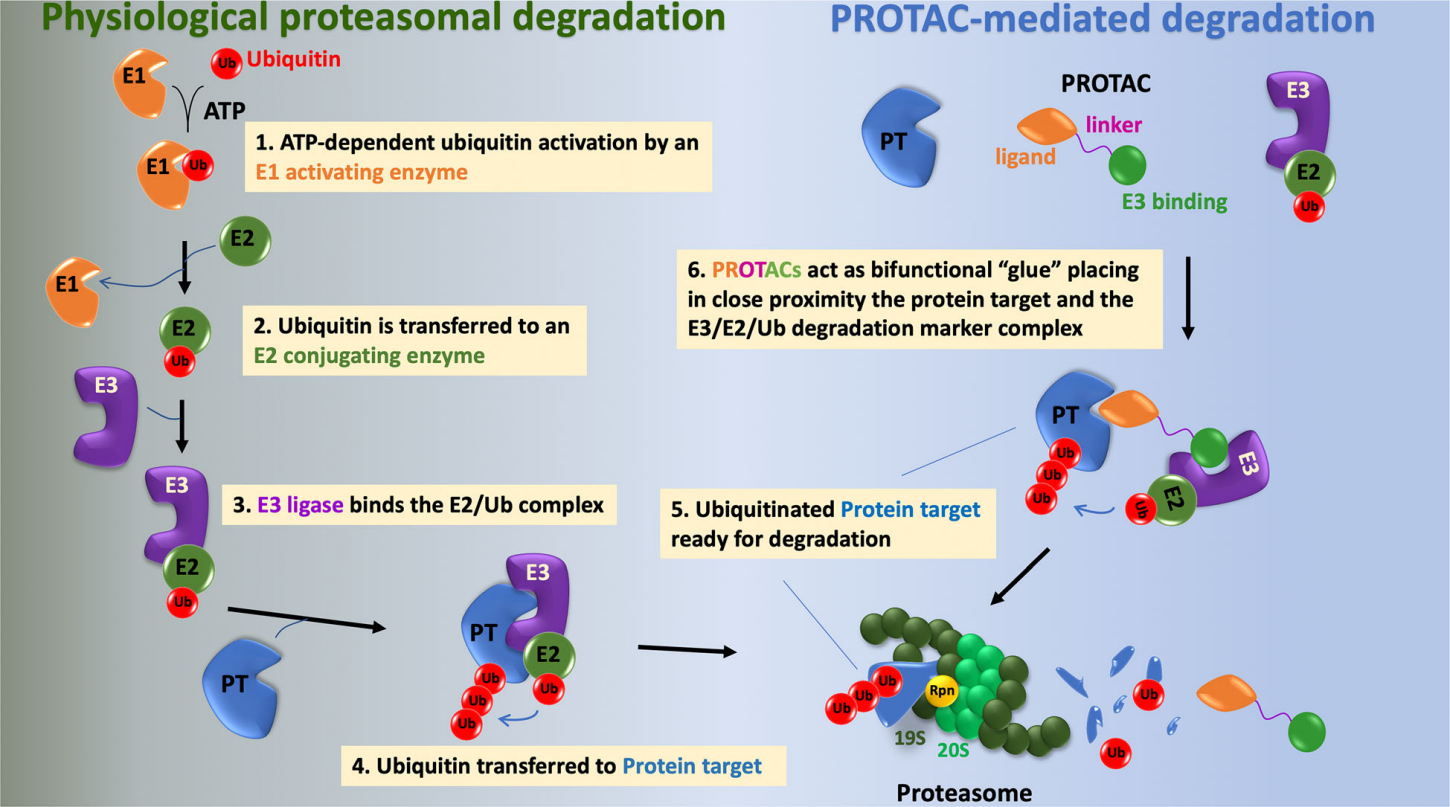
The ubiquitin-proteasome system and PROTACs. The left part of the figure shows the relevant steps in the tagging of proteins for degradation by the ubiquitin-proteasome system. That process involves sequential steps catalyzed by three types of enzymes. The E1 activating enzyme catalyzes the activation of ubiquitin in an ATP-dependent process. The active site cysteine present in E1 established a bond with the carboxy-terminus of ubiquitin. In a second step, the thioesterified ubiquitin is transferred to the E2 ubiquitin conjugating enzyme. In a third step, the E3 ligase binds both the protein target and the E2-ubiquitin. The E3 ligases are the most numerous (more than 500) and are expected to contribute to the specificity in the degradation of the protein target. On the other side, only two E1 have been described and forty E2. The E3-E2-ubiquitin-Protein target multiprotein complex is then competent to transfer ubiquitin to lysine residues of the protein target. The ubiquitinated protein can then be targeted to the proteasome for degradation. Rpn receptors present in the 19S unit of the proteasome help degradation of tagged proteins acting as binding sites. Proteins entering the proteasome are then degraded by peptidases of the 20S region, resulting in the formation of fragmented proteins and the removal of the ubiquitin from the protein being degraded. The right part of the figure illustrates the mechanism of action of PROTACs. These molecules are heterobifunctional constructs consisting in a ligand that specifically binds the protein target and an E3 binding molecule. A linker is necessary to connect both the ligand and the E3 binding molecule. PROTACs act by stabilizing in close proximity the protein target and the E3-E2-Ubiquitin complex. That ternary complex (PROTAC+protein target+E3-E2-Ubiquitin) allows ubiquitination of the protein target, that is then recognized for degradation by the proteasome. PROTACs, therefore, take advantage of the protein degradation system to direct the removal or down regulation of a protein target that may play a pathophysiological role in a disease. In this respect, adequate engineering of a PROTAC may favor degradation of pathophysiological proteins in a cell or tissue-specific manner, for example, by directing degradation by E3 ligases specifically or mainly present in leukemic blasts or nervous tissue

Proteasomal-mediated protein degradation is an ordered multistep and sequential process, which requires several enzymatic reactions [16]. Ubiquitination is produced by three different steps: (i) activation by an E1 Ub-activating enzyme, (ii) conjugation mediated by the E2-conjugating enzyme, and finally (iii) ligation that is produced by the E3-protein ligases (Fig. 1) [17, 18]. In mammalian cells there are two E1 that can bind to forty E2s, which can bind with hundreds of E3s in a hierarchical way [19]. In the activation process, E1 enzymes bind both ATP and ubiquitin and catalyse the acyl-adenylation of the C-terminus of the ubiquitin molecule and transfer ubiquitin to a cysteine residue producing a thioester linkage between the C-terminal carboxyl group of ubiquitin and the sulfhydryl group of the E1 cysteine [17, 18]. E1-thioesterified ubiquitin is then ready to transfer the latter to a cysteine located in the active center of an E2 conjugating enzyme [20]. Finally, in the ligation process, E3 ligases act bridging the E2-ubiquitin and the substrate and create an isopeptide bond between a lysine of the target protein and the C-terminal glycine of ubiquitin. This last step provides the substrate specificity of the reaction as there are hundreds of E3 ligases, being the majority of them included in two families”: the HECT and the RING ligases [21, 22]. In addition, these ligases vary depending on cellular and tissues contexts, diversifying their protein substrates [23]. Rpn receptors present in the 19S unit of the proteasome help degradation of tagged proteins acting as binding sites [23]. Proteins entering the proteasome are then degraded by peptidases of the 20S region, resulting in the formation of fragmented proteins and the removal of ubiquitin from the protein being degraded [23].

## Development of PROTACS

PROTACs take advantage of the ubiquitin-mediated degradation system as a therapeutic strategy. A PROTAC is a molecule that consists of three parts: (i) a ligand (warhead) that interacts with the protein to be degraded, (ii) a different ligand that binds to an E3 ubiquitin ligase and (iii) a linker that connects both ligands (Fig. 1) [5]. The proximity between the E3 ligase and the protein target achieved by the heterobifunctional PROTAC facilitates ubiquitination and degradation of the protein target. First PROTAC compounds were developed more than 20 years ago using the E3 ligase TRCP [24]. Of note in this work the phosphopeptide ligand for TRCP did not penetrate the cellular membrane, limiting their development [24]. That aspect merits consideration as membrane permeation still represents a limiting step in the development of PROTACs. Later on, peptide ligands were changed to small molecules to avoid the low potency and to increase the cell permeability [25]. Nutlin-3a a ligand of the E3 ligase MDM2 was then used, showing capacity to degrade the androgen receptor [25].

Several structural studies have helped in the development of PROTACs. For instance, Van Molle and colleagues used a fragment based lead discovery approach to identify regions in VHL that are used for its interaction with the target protein hypoxia inducible factor1α (HIF1α) [26]. Crystal structures revealed a site of interaction of VHL with a 19 amino acid peptide derived from HIF1α. In silico and structural analyses identified three drugs that bound to the same site in VHL as the HIF1α peptide and acted as competitors of that protein-protein interaction [26, 27]. An ulterior study in the same experimental setting allowed development of drugs with better membrane permeabilization properties [28]. The above studies allowed identification of the groove on the surface of pVHL that was used to interact with a region of HIF1α, paving the way to the development of VHL-based PROTACs.

An important effort was made to develop warheads to target the nuclear hormone receptors AR and ER, generating a series of PROTACs which showed preclinical activity [29–34]. Other warheads that were developed later included known tyrosine kinase inhibitors, or novel families of epigenetic agents like the Bromo and Extraterminal Domain inhibitors (BETi) [31, 33]. These proteins belong to super enhancer complexes that regulate the expression of TFs, indirectly modulating transcription initiation and elongation [35]. Most studies evaluating BET-PROTACs have been performed in leukemia and lymphoma, followed by some indications in solid tumors like prostate cancer, triple negative breast cancer or osteosarcoma (Table 1). BETi provided a therapeutic opportunity to target transcription, especially due to the difficulties in targeting TFs, which were considered as undruggable targets [43]. BETi have shown antitumoral activity in several haematological malignancies and solid tumors [43, 44], and BET-PROTACs were developed with the aim to boost and prolong the pharmacological effect of these agents, increasing their anti-tumoral activity. Beyond the activity of BET-PROTACs in different solid and hematologic tumors, these agents have also demonstrated activity in preclinical models that were resistant to BETi, suggesting that resistant tumors still depend on these proteins [38].

**Table 1:** Reported studies evaluating BET-PROTACs

Transcription factor	Ligand for E3 ligases	Cancer type	BET Inhibitor	PROTAC	Reference
BRD4	Von Hippel-Lindau (VHL) E3 ligase, E3 ubiquitin ligase CRBN	Osteosarcoma, leukemia	JQ1	BETd-260	[36]
BRD4	E3 ubiquitin ligase CRBN	Leukemia, Burkitts Lymphoma	Oxazepines, JQ1, OTX	QCA570, ARV-825	[34, 37]
BRD1, BRD2 and BRD4	E3 ubiquitin ligase CRBN	Burkitt’s lymphoma	JQ1, OTX	ARV-825	[34]
BRD4 over BRD2 and BRD3	Von Hippel-Lindau (VHL) E3 ligase	Cervical carcinoma	JQ1	MZ1	[33]
BRD4	Von Hippel-Lindau (VHL) E3 ligase	Triple negative Breast Cancer and JQ1 resistant cells	JQ1	MZ1	[38]
			OTX	ARV-825	
BRD2, BRD3 and BRD4	E3 ubiquitin ligase CRBN	Leukemia	JQ1	dBET1	[31]
BRD2, BRD3 and BRD4	E3 ubiquitin ligase CRBN	Triple negative Breast Cancer	BETi-211	BETd-246	[39]
Pan BET degrader	Von Hippel-Lindau (VHL) E3 ligase	Prostate cancer	BETinhibitor	ARV-771	[40]
Bromodomain containing proteins: BRD9	E3 ubiquitin ligase CRBN	AML	BRD9 inhibitor	dBRD9	[41]
Bromodomain containing proteins: BRD7/BRD9	Von Hippel-Lindau (VHL) E3 ligase	Leukemia	BRD9/BRD7 inhibitor	VZ185	[42]

Additional PROTACs targeting other components of the transcription machinery include those based on inhibitors of CDK9 or SMARCA2/4 [45–49]. PROTACs based on inhibitors targeting ALK or CDK6 kinases or based on inhibitors of the BCL6, BCL-XL or MCL1 apoptotic machinery components have also been developed [50–55]. Supplementary Table 2 shows a complete list of PROTACs explored in preclinical studies.

**Fig. 2:**
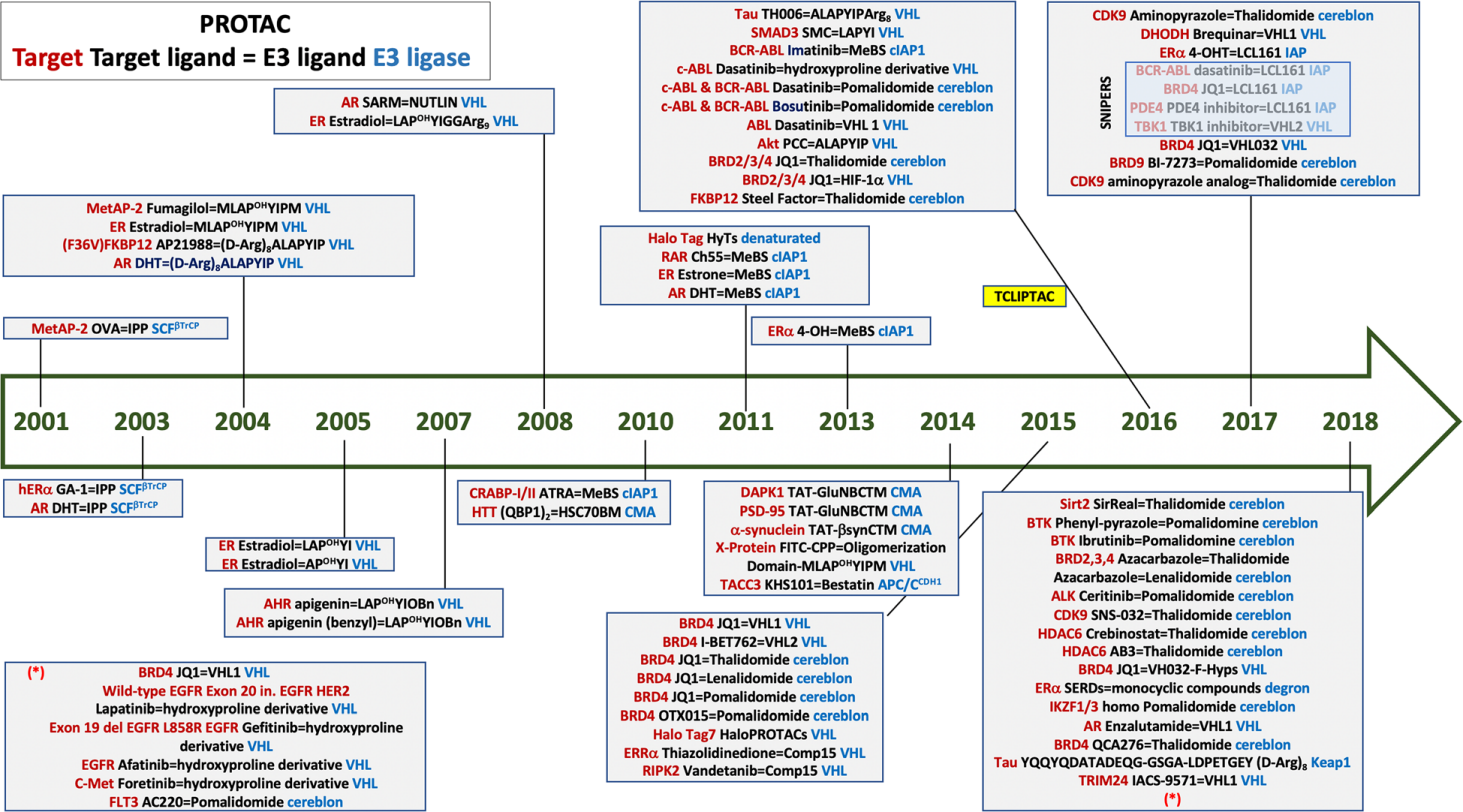
Chronological representation of all different types of PROTACS since 2001 including the structure and type of ligase. Information about the type of warhead is also included particularly for the most recent compounds

Although many of these agents have been designed and evaluated preclinically, only two PROTACs, ARV-110 and ARV-471, have entered the clinical setting. ARV-110, a PROTAC designed to provoke degradation of the AR is being analysed in patients with castration resistant metastatic prostate cancer who have progressed on at least two prior therapies (enzalutamide or abiraterone, see clinicaltrials.gov reference NCT03888612). ARV-471, that provokes the degradation of the ER, is being explored in ER positive locally advanced or metastatic breast cancer (NCT04072952) [9]. Figure 2 describes all the chronological process for the development of this family of agents including information for each compound.

## Optimizing PROTACs

There is still much room to optimize this family of relatively new agents. For instance, it is relevant to mention that only 1% of the more than 500 E3 ligases have been explored for target degradation, and the selection of the E3 pairing seems to be critical [56]. Indeed, E3 ligases dictate target specificity [56]. Only few ligases have been explored, including CRBN, VHL, IAPs, MDM2, DCAF15, DCAF16 and RNF114, but only CRBN and VHL have shown activity in preclinical models [56]. Adequate optimization of the selection of the E3 ligases may increase efficacy and decrease potential toxicity of the PROTACs. One opportunity is based in their tissue specific expression. Thus, ASB9, a SOCS box E3 ligase, is only expressed in pancreatic and testis tissue, and FBXL16 is mainly found in the cerebral cortex, providing the possibility of acting on diseases affecting those tissues [56]. Similarly, if a ligase is only expressed in specific cellular components it can be used to degrade proteins located at that particular cellular site, as is the case for the use of the nuclear E3 ligase DCAF16 for nuclear targets [57]. Another example is represented by the action of CRBN against Ikaros and Aiolos, both nuclear proteins [3, 56].

Other parameters beyond the mere presence of the E3 ligase have to be taken into consideration when evaluating how active a PROTAC can be. Thus, ideally, the interaction between the ligand for the protein of interest and the E3 ligase should promote the formation of ternary complexes (Protein of interest-PROTAC-E3 ligase), leading to polyubiquitination of the protein and subsequent proteasomal degradation. However, in some circumstances such ternary complex may fail to be produced. That may happen, for example, in case the concentration of the PROTAC is in large excess with respect to the E3 ligase and the protein target. Since PROTACs are bifunctional molecules, they may independently bind to two molecules: the E3 ligase and the protein target. Desirably, one PROTAC molecule would act as a bridge between one E3 ligase and one protein target, creating a ternary complex. In case high concentrations of the PROTAC are present, it is possible the formation of binary complexes (protein target-PROTAC, or E3 ligase -PROTAC) that are ineffective [58]. In this circumstance, the right equilibrium is not achieved, since elevated PROTAC concentrations would saturate binding sites on the E3 ligase on one side and on the protein target on the other side, exhausting free forms of these proteins that could be used for ternary complexes. This process, produced when high concentrations of the PROTAC are present, is called the “hook effect” [58, 59]. It is relevant to mention that some PROTACs such as MZ1 may mitigate the hook effect as they exhibit positive cooperativity with respect to the assembly of ternary complexes.

The chemical characteristics of the linker can also affect the degradation capacity of the PROTAC. For instance the linker length can modify the degradation profile of lapatinib-based PROTACs targeting the EGFR and HER2 or only EGFR [60]. A similar finding was observed when different linkers were developed tethering JQ1 to VHL-1 showing that some PROTACs were able to degrade BRD2–4 and others were specifically selective for BRD4 [33].

## Exploiting the use of PROTACS in the clinical setting

### Inhibition versus degradation

In the case of certain proteins, especially those with enzymatic activity, PROTACs can have a double mechanism of action. In fact, PROTACs based on inhibitors of the kinase activity of a protein should retain the beneficial properties of inhibiting the kinase in addition to the capability of the PROTAC to reduce the amount of the protein kinase. These two effects sum to achieve an even greater inhibitory action on the protein kinase, as compared to the mere inhibition of the kinase activity. In fact, PROTACs can potentially be more effective as they induce target degradation rather than solely target inhibition and the effect can be prolonged as it depends on the re-synthesis rate of the inhibited protein [3, 32, 61–65]. Some recent examples have demonstrated that low affinity warheads can induce degradation of targets of interests, being more efficient that just their chemical inhibition, as has been demonstrated for p38 [66]. In that report, the best predictor of efficacy was ternary complex formation. Indeed, of the 54 kinases inhibited by the kinase inhibitor foretinib, 14 were degraded by the CRBN-based PROTAC and 9 by the VHL PROTAC and six by both [66].

A limitation for all drug modalities that target proteins including PROTACs is how much protein is needed to be degraded to induce a biological effect. However, degraders, as a catalytic modality, are troubled less by this issue as they do not depend on receptor occupancy. Another aspect that requires refinement is the elucidation of the most adequate competent poly-ubiquitination process to mediate the effect. Lack of activity of a recent described PROTAC with an inhibitor of *KRAS* as a warhead was explained by the limitation of the poly-ubiquitination process due to the electrostatic interactions produced by the poly-lysines in the C-terminus of *KRAS* [67]. Indeed, in some cases ubiquitination of the target does not occur [64].

### Reducing clinical toxicities

It is considered that PROTACs could be potentially toxic due to several reasons. The first one is that if the targeted protein is widely expressed in non-transformed tissues, its degradation can produce serious on-target side effects when applied to patients [5]. However, several strategies could be used to reduce this problem. As described before E3 tissue specificity can be incorporated to reduce on target dose-limiting toxicities. A recent example of this has been described with the development of a BCL-XL PROTAC. BCL-XL inhibitors were not approved for the treatment of B cell lymphoma due to its on-target and dose limiting toxicity, mainly thrombocytopenia [53]. Since the VHL E3 ligase is poorly expressed in platelets, BCL-XL PROTACs targeted for degradation by that ligase do not induce thrombocytopenia, maintaining the same therapeutic efficacy as VHL is expressed in the lymphomatous cells [68]. In a similar way, presence of ligases in specific tissues can increase the activity in those places reducing the toxicities in other cells [56]. It is therefore expected that an appropriate selection of the target protein and the E3 ligase will not only increase specificity, but augment effectiveness and reduce side effects.

Another strategy to reduce toxicity is the vectorization of these compounds with antibodies so the compound can specifically reach the tumoral cell. This can be done creating a PROTAC-ADC or with the incorporation of PROTACs into nanoparticles that can secondarily be vectorised with antibodies [68–70]. A proof of concept example of this approach is the report of an ADC by attaching a BET degrader to an anti-CLL1 antibody [71].

### Clinical implications: overcoming mechanisms of resistance

A classical mechanism of resistance to kinase inhibitors is the presence of primary or secondary mutations in the kinase domain that decrease or prevent the binding of the compound in the ATP pocket. For instance, mutations in Brutons tyrosine kinase are involved in resistance to ibrutinib, an inhibitor of this kinase that is approved for the treatment of several haematological malignancies such as relapse/refractory mantle cell lymphoma, chronic lymphocytic leukemia and Waldenström macroglobulinemia [72, 73]. Analogously, it is well known that mutations in the chimeric oncogene *BCR/ABL* cause resistance to tyrosine kinase inhibitors used in chronic myeloid leukemia [74]. In the case of solid tumors, mutations in the EGFR, such as the T790M have been associated to resistance to first generation EGFR kinase inhibitors such as gefitinib or erlotinib [74]. For these diseases in which tyrosine kinases play a pathophysiological role, development of PROTACs with a warhead able to bind the mutated kinase, for example at an allosteric site, could result in a stable interaction potentially rescuing the resistance [75]..

Resistance due to mutations in proteins which lack enzymatic activity can also be bypassed by PROTACs. Thus, PROTACs targeting the ER in breast cancer could rescue resistance to anti-estrogens when this resistance is mediated by mutations at the ER, supporting the development of ER PROTACs in this situation [76]. Moreover, in prostate cancer AR PROTACs have demonstrated more efficacy than enzalutamide in castration resistance prostate cancer, opening the door to the development of AR-PROTACs in the clinic [40, 77].

### Mechanisms of resistance to PROTACs

Several studies indicated that genomic alterations affecting protein integrity of components of the ubiquitin-proteasome system may be behind resistance to PROTACs. Loss of E2 or E3 ligases or the cullin (CUL) proteins have been implicated. Zhang and colleagues observed that resistance to CRBN-based BET-PROTACs was provoked by chromosomal deletion of the *CRBN* gene [78]. On the other hand, resistance to VHL-based BET-PROTACs was found to occur by cullin-2 (CUL2) loss of function due to several genomic alterations in the *CUL2* locus, including exon 12 skipping or frameshift mutations which gave rise to a premature stop codon [78]. Similar findings were also observed by Ottis et al. [79]. Using RNAi of components of the ubiquitin-proteasome system in cells made resistant to BET-PROTACs confirmed that down-regulation or loss of those proteins may lead to PROTAC resistance [79]. Those authors also identified the COP9 signalosome as implicated in the function of BET-PROTACs. Using a CRISPR/Cas9 screen to define effectors involved in targeted protein degradation, Mayor-Ruiz and colleagues confirmed a role of the COP9 signalosome and CUL proteins in the regulation of targeted protein degradation [80]. Of note, no molecular alterations were observed in the proteasome or in the binding of the ligands to protein target or the E3 ligases.

## Concluding remarks

Exploitation of the protein degradation machinery for therapeutic purposes opens new possibilities to target proteins involved in pathophysiological processes. Although important advances have been made using PROTAC technology, there are still many challenges for their clinical development. A crucial aspect is the selection of proteins which play a major oncogenic role in a certain tumor type, as is the case of the AR in prostate cancer or the ER in breast cancer. Optimization of the ligases used in the design of PROTACs for specific tumor tissues or cell types, or vectorization of the compounds with specific antibodies are strategies to be implemented and exploited. In addition, selection of the best combination with other therapies could reduce side effects augmenting activity. PROTACs are not limited to cancer therapy and they are under investigation in all diseases where an accumulation of proteins are important in their pathogenesis. That is the case in some neurodegenerative diseases or in conditions where degradation of a protein could have major impact than its enzymatic inhibition, as in the case of IRAK4 targeting in autoimmune diseases [81, 82]. Finally in situations where the target has a scaffolding role that cannot be inhibited by a conventional inhibitor or forms part of a hard-to-drug target, PROTACs could play a central role [82]. In conclusion, the first steps have been taken and offer hope for the incorporation of this family of agents in the clinic.

## Supplementary information

**Additional file 1: Supplementary Table 2.** Reported studies describing PROTACs [45–49, 52–55, 60, 65, 66, 75, 77, 83–91].

## Data Availability

Data are available upon reasonable request to the corresponding author.

## Abbreviations

AR: Androgen receptor is a nuclear receptor activated by androgenic hormones, including testosterone and dihydrotestosterone
BET: Bromo and Extraterminal Domain is a protein domain that recognizes acetylated lysine residues. Members of this family include BRD2, BRD3, BRD4 and BRDT
BTK: Brutons tyrosine kinase is a tyrosine kinase encoded by the *BTK* gene in humans. Mutations in this kinase are associated with haematologic malignancies.
CRBN: Cereblon is a protein that in humans is encoded by the *CRBN* gene. Cereblon forms an E3 ubiquitin ligase complex that is involved in the ubiquitination of several proteins. It is the target of immunomodulatory IMiDs
IAP1: Inhibitor of apoptosis protein 1(also named BIRC2), codes for the cellular inhibitor of apoptosis protein-1 that is involved in the ubiquitination of the pro-survival kinase TAK1
DUBs: Deubiquitinating enzymes are a group of proteases that cleave ubiquitin from proteins, therefore avoid the degradation of proteins via the proteasome
IMiD: Immunomodulatory drugs include thalidomide, lenalidomide or pomalidomide
PROTACs: They consist of two linked molecules: one capable of engaging an E3 ubiquitin ligase, and another that binds to a target protein that is of interest for proteasomal degradation
TFs: A class of proteins that control, by binding to a certain DNA sequence, the transcription of genetic information from DNA to messenger RNA
