# Supplementary material for: Proteolysis targeting chimeras (PROTACs) in cancer therapy
Source: J Exp Clin Cancer Res. 2020 Sep 15;39:189. doi: 10.1186/s13046-020-01672-1 (PMC7493969; doi:10.1186/s13046-020-01672-1)
Supplement: Supplementary file 1 — Additional file 1: Supplementary Table 2. Reported studies describing PROTACs [45–49, 52–55, 60, 65, 66, 75, 77, 83–91]. [file 13046_2020_1672_MOESM1_ESM.docx]

**Supplementary table 2. Reported studies describing PROTACs**

| **Target Protein** | **Type of target** | **Warhead** | **Ligand for E3Ligases** | **Preclinical tumor model: *in vitro* or *in vivo* data** | **Reference** |
| --- | --- | --- | --- | --- | --- |
| ALK |  |  | Von Hippel-Lindau (VHL) E3 ligase | ALK-positive NSCLC ALK fusion positive cell lines, SU-DHL-1 and H3122  H3122 xenograft model | 50,51 |
|  | Protein kinase | Ceritinib |  |  |  |
|  |  |  |  |  |  |
|  |  |  |  |  |  |
|  |  |  |  |  |  |
| SMARCA2 SMARCA4 | Transcriptional Regulator |  | Von Hippel-Lindau (VHL) E3 ligase | Myeloid leukemia (AML) Acute myeloid leukemia cells | 49 |
| BCL6 | Transcriptional regulator |  | E3 ubiquitin ligase CRBN | Diffuse large B-cell lymphoma (DLBCL)  Weak antiproliferative response | 52 |
| BCL-XL | Apoptotic mediator | ABT263 | Von Hippel-Lindau (VHL) E3 ligase | Leukemia and cancer cells Several xenograft tumors | 53 |
| BET  BRD4  pan-BET | Transcriptional regulator | OTX015 | E3 ubiquitin ligase CRBN: ARV-825    E3 ligase VHL: ARV-771 | Post-myeloproliferative neoplasm secondary (s) AML cells,  Patient-derived cells,  in vitro generated ruxolitinib-persister or ruxolitinib-resistant sAML cells,  In vivo sAML mouse model | 83 |
| Dual  PLK1/BRD4 | Mitotic kinase/Transcriptional regulator | BI2536 | CRBN E3 ligase-based BET PROTAC | Human acute leukemia MOLM-13 and KG1 cells, MV4-11 tumor xenograft model | 83 |
| CDK6 | Protein kinase | Palbociclib | E3 ubiquitin ligase CRBN | Hematopoietic cancer cells including multiple myeloma and copy-amplified/mutated forms of CDK6 | 54 |
| CDK6 | Protein kinase | Palbociclib | E3 ubiquitin ligase CRBN | Acute myeloid leukemia cells | 55 |
| CDK6 | Protein kinase | Palbociclib | E3 ubiquitin ligase CRBN | Pancreatic cancer | 55 |
| CDK9 | Protein kinase | Wogonin (natural product isolated from the *Scutellaria baicalensis*) | E3 ubiquitin ligase CRBN | Breast cancer | 45 |
| CDK9 | Protein kinase |  | E3 ubiquitin ligase CRBN | HCT116 cells. No explored anti-proliferative activity | 46 |
| ERα | Transcriptional regulator |  | Von Hippel-Lindau (VHL) E3 ligase | ERα-positive breast cancer cells, MCF-7 mouse xenograft model | 47 |
| ER | Transcriptional regulator | ERD-308 | CRBN ligand/cullin 4A system  VHL ligand/Cullin 2 | MCF-7 and T47D ER+ breast cancer cell lines | 47 |
| MCL1 | Apoptotic mediator |  | E3 ligase CUL4A−DDB1 CRBN E3 ligase | Acute myeloid leukemia | 48 |
| SGK3 | Protein kinase | SGK inhibitors 308-R or 290R | E3 ubiquitin ligase CRBN | ZR-75-1 and CAMA-1 cancer cell lines | **84** |
| STAT3 | Transcriptional regulator | SI-109 and SI-108 | E3 ubiquitin ligase CRBN  Von Hippel-Lindau (VHL) E3 ligase | Acute myeloid leukemia and anaplastic large-cell lymphoma cell lines, multiple xenograft mouse models | **85** |
| MEK1/2 | Protein kinase | MS342 | Von Hippel-Lindau (VHL) E3 ligase or  E3 ubiquitin ligase CRBN | A375 cells | **86** |
| FLT-3 | Protein kinase | Quizartinib |  | Acute myeloid leukemia | **87** |
| MET  and  VEGFR | Protein kinase | Foretinib | Von Hippel-Lindau (VHL) E3 ligase | MDA-MB-231 cell lines Triple negative breast cancer | **66** |
| FAk | Protein kinase | Defactinib |  | Breast cancer cell lines | **65** |
| AR | Transcriptional regulator | Enzalutamide | Von Hippel-Lindau (VHL) E3 ligase | Prostate cancer cell lines: VCaP, LNCaP, PC3. | **77** |
| BTK | Protein kinase | Ibrutinib | IAP or cereblon E3 ligases |  | **75** |
| EGFR/MET | Protein kinase | Lapatinib, gefitinib, afatinib, foretinib | Von Hippel-Lindau (VHL) E3 ligase | Cell lines: OVACR8, HeLa, HCC827, H3255, H1975, MDA-MB-231 | **60** |
| SGK3 and PIK3C3 | Protein kinase | Chloroalkane | Cul2  Von Hippel-Lindau (VHL) E3 ligase VHL and  cIAP ligands | Kidney cancer | **88** |
| c-ABL and  BCR-ABL | Protein kinase | Bosutinib and dasatinib | CRBN or VHL E3 ligases | K562 human chronic myelogenous leukemia cells | **89** |
| B-Raf | Protein kinase |  | CRBN E3 ligases | Breast cancer cell line: MCF7 | **90** |
| MDM2 | E3 Protein ligase | MD-224 | CRBN ligands thalidomide and lenalidomide /cullin 4 E3 ligase complex | Leukemia cell lines, RS4;11 xenograft tumor model | 91 |
